# Training drills in high performance badminton—effects of interval duration on internal and external loads

**DOI:** 10.3389/fphys.2023.1189688

**Published:** 2023-06-30

**Authors:** Antonia Edel, Jan-Luka Weis, Alexander Ferrauti, Thimo Wiewelhove

**Affiliations:** ^1^ Department for Training and Exercise Science, Faculty of Sports Science, Ruhr University Bochum, Bochum, Germany; ^2^ Department of Fitness and Health, IST University of Applied Sciences, Düsseldorf, Germany

**Keywords:** on-court training, elite level badminton, training load, physiological responses, multifeeding

## Abstract

**Purpose:** The aim of the present study was to analyze the impact of interval duration on training loads and technical skill performance in high performance badminton drills.

**Methods:** On three experimental days, 19 internationally ranked players (13 male: 22.7 ± 3.8 years, 180 ± 6 cm, 71.5 ± 6.1 kg; 6 females: 20.4 ± 2.5 years, 168 ± 4 cm, 59.8 ± 6.0 kg) completed one of three protocols (T_10_, T_30_, and T_50_) of a typical badminton specific drill, the so-called “Multifeeding” (the coach feeds shuttlecock without break in a random order) in a counterbalanced order. The protocols varied in interval duration (10, 30, and 50 s) but were matched for the rally-to-rest-ratio (1:1) and active playing time (600 s). Cardiorespiratory responses (portable spirometry, chest belt), energy metabolism (levels of blood lactate, La), rate of perceived exertion (RPE), player’s kinematics (Local Positioning System), and technical skill performance (video analysis) were measured.

**Results:** Average oxygen consumption (T_10_ 45 ± 6; T_30_ 46 ± 7; T_50_ 44 ± 6 mL min^−1^·kg^−1^), Energy expenditure (886 ± 209; 919 ± 176; 870 ± 206 kcal h^−1^), heart rate (164 ± 13; 165 ± 11; 165 ± 10 bpm) and RPE (16 ± 2; 17 ± 2; 17 ± 2) did not differ between the protocols. Respiratory exchange ratio (RER) and La significantly increased depending on interval duration (RER: 0.90 ± 0.05; 0.93 ± 0.03; 0.96 ± 0.04 and La: 3.6 ± 2.0; 5.6 ± 3.0; 7.3 ± 2.3 mmol l^−1^). Stroke frequency (SF; 0.58 ± 0.05; 0.57 ± 0.05; 0.55 ± 0.06 strokes·s^−1^) was similar while distance covered, and average running velocity were significantly lower for T_50_ compared to T_10_ (76 ± 17; 70 ± 13; 65 ± 11 m min^−1^). Moreover, jump frequency in T_30_ was higher than in T_10_ (6.7 ± 3.1; 8.8 ± 3.8; 8.5 ± 4.2 jumps·min^−1^), whereas differences in internal and external loads were not associated with changes in stroke precision (errors: 16 ± 6; 19 ± 4; 18 ± 4%; accuracy: 22 ± 6; 24 ± 8; 23 ± 8%).

**Conclusion:** Anaerobic metabolic stimulus increases while running distance and velocity decrease, in case of longer interval durations. Even though there was no impact on stroke precision, extending the intervals beyond 30 s might impair external training load and skill performance. Consequently, interval duration should be defined carefully depending on the training goals.

## 1 Introduction

Badminton match play is characterised by a highly intermittent structure, where short bouts of high-intensity work (about 7 s) alternate with rest periods approximately twice as long (about 15 s) over an extended period of time (between 17 min and 1 h) (Phomsoupha and Laffaye, 2015; Edel et al., 2019). The short bouts of high-intensity work (strokes, quick changes of direction, rapid accelerations, and decelerations) require high levels of quickly available energy. These are mainly provided by anaerobic metabolic pathways, such as alactic intramuscular phosphates [particularly adenosine triphosphate (ATP) and creatine phosphate (CrP)] and anaerobic glycolysis. As alactic anaerobic sources are depleted after a few seconds and the use of lactic pathways over an extended period of time lead to accumulated acidosis, players rely on the rest periods to ensure active recovery. In this regard, a high aerobic capacity is important for restoring the limited anaerobic energetic sources as well as for lactate elimination during these rest periods (Faude et al., 2007; Fernandez-Fernandez et al., 2012). Consequently, elite players need both, a well-developed anaerobic but also high aerobic endurance capacities to achieve high levels of performance.

Therefore, badminton-specific endurance training should focus on the ability to repeatedly perform short bouts of high-intensity work but also on rapid recovery. At the same time, also high quality of performance outcomes (e.g., low error rates and accurate attacking shots) must be maintained to meet the complex requirements of the game. However, neuromuscular fatigue may impair the quality of these performance outcomes. In this context, previous studies in other racquet sports have shown that small changes in training prescription can considerably change the internal and external training loads and influence performance outcomes. For instance, a study on tennis showed that reducing rest periods from 15 to 10 s in multiple sprint and stroke combinations led to significantly lower running and stroke velocities (Ferrauti et al., 2001a; Ferrauti et al., 2001b). Another study, also on tennis, revealed that prolonging the interval duration from 30 to 60 s resulted in decreased stroke velocity and stroke precision (Reid et al., 2008). Concludingly, the choice of the training prescription is essential for the training outcomes.

However, research regarding on-court training is rare, and to the best of our knowledge, only one study has considered the physiological outcomes of on-court training drills in badminton, so far. This study measured heart rate (HR) and levels of blood lactate (La) during multifeeding (a typical badminton-specific endurance drill) training and compared it to the physiological responses of competitive match play (Majumdar et al., 1997). According to this study, players reached significantly higher HR and La during the multifeeding drill compared to match conditions. Majumdar et al. (1997) warned that high La accumulation can negatively influence coordination, performance outcomes, and, consequently, the quality of training drills. However, they did not evaluate performance outcomes, consider the impact of variations in training prescription, or make connections to predetermined training goals. Therefore, the impact of training prescription on internal and external training loads and their impact on performance outcomes in badminton remains unclear.

As a result, in current practice, coaches select training protocols with interval durations ranging from a few seconds to several minutes, regardless of scientific evidence for training outcomes and efficiency. This seems problematic, especially in a sport characterised by high frequency of numerous worldwide competitions that reduce overall training time. To overcome this problem and provide more evidence for goal directed training prescription, the aim of the present study was to clarify the impact of interval duration on internal and external training loads and performance outcomes in high-performance badminton drills.

## 2 Materials and methods

### 2.1 Participants

Nineteen internationally ranked badminton players (13 male, 22.7 ± 3.8 years, 180 ± 6 cm, 71.5 ± 6.1 kg, world ranking positions between 11 and 386; 6 female, 20.4 ± 2.5 years, 168 ± 4 cm, 59.8 ± 6.0 kg, world ranking positions between 22 and 536) participated in this study. All players were members of the German Olympic, perspective, or junior squad and were trained in one of two training groups at the federal base for badminton singles. To determine their anaerobic threshold and maximal HR (HR_max_), players performed an exhaustive stepwise incremental test on a treadmill. The anaerobic threshold was defined as a La concentration of 4 mmol l^−1^ (Wackerhage et al., 2022). Table 1 shows the participants’ anthropometric data and performance characteristics. Before the study started, the players, their coaches, and their guardians (in the case of minors) were informed about the testing procedures, data policy, and potential risks of the study, and gave their written informed consent to voluntarily participate. The players were instructed to maintain a regular diet and to perform no additional vigorous exercise prior to the evaluation. Since their weekly training structures were comparable, players were allowed to continue their usual training routines before and after the experimental days but were excluded from testing in the weeks prior to or following international tournaments. The study design, procedures, and measurements aligned with the Declaration of Helsinki and were approved by the local ethics committee of the Faculty of Sports Science at Ruhr University, Bochum (EKS V 21/2019).

**TABLE 1:** Anthropometric data and performance characteristics of the participants.

	N	Age [years]	Height [cm]	Bodyweight [kg]	v_4mmol_ [m·s^−1^]	HR_4mmol_ [bpm]	HR_max_ [bpm]
Male	13	22.7 ± 3.8	180 ± 6	71.5 ± 6.1	4.4 ± 0.4	179 ± 34	189 ± 9
Female	6	20.4 ± 2.5	168 ± 4	59.8 ± 6.0	3.9 ± 0.2	177 ± 14	181 ± 1

Data are shown as mean ± standard deviation; N, number of subjects; v_4mmol_, velocity at lactate concentration of 4 mmol during incremental treadmill test; HR_4mmol_, Heart rate at lactate concentration of 4 mmol l^−1^ during incremental treadmill test; HR_max_, maximum heart rate during incremental treadmill test.

### 2.2 Design and procedures

**FIGURE 1:**
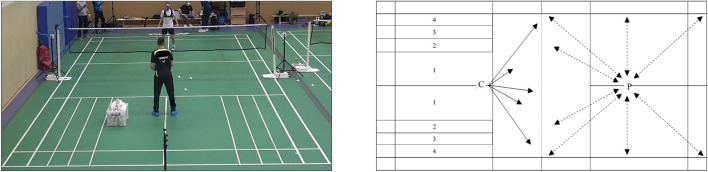
Set up of the Multifeeding drill. *Left*: Badminton court with the markers for hitting zones, coach and player are standing at their starting positions. *Right*: schematic illustration of the set up with the hitting zones (1–4 from inner to outer field), Zone 4 was defined as target zone. *C* position of the coach that feeds the shuttlecocks, *P* starting position of the player, unbroken arrows illustrate the shooting direction of the feeds, pointed arrows show possible running paths of the player.

Based on a cross-sectional study design, each player engaged in one of three multifeeding training protocols (T_10_, T_30_, and T_50_) on three different training days. Multifeeding on-court drills are typically used to improve badminton-specific endurance. In this drill the coach feeds shuttlecocks with no breaks and in random order from the centre of one side of the net to the player on the other side of the net while the player aims to reach and return each ball in a preferably high quality of strokes (velocity and accuracy) (Figure 1). The rally-to-rest ratio (1:1) was identical, but training protocols varied in interval and rest duration (10, 30, and 50 s). Number of sets and repetitions were adjusted to ensure an identic total playing time (600 s). The details of the training protocols are shown in Table 2. To quantify stroke accuracy, markers on the ground divided each side of the back field into four hitting zones (Figure 1). Players were advised to place the shuttlecocks in the outer hitting zone (zone 4), that was defined as the target zone, whenever they performed an attacking shot (usually a smash). During the session, portable spirometry and chest-belt measurements monitored cardiorespiratory responses. Blood samples were taken to measure La, and players were asked for rate of perceived exertion (RPE) at predefined time points. Moreover, player kinematics were monitored via a local positioning system (LPS), accelerometery, and video analyses, and performance outcomes were evaluated via video analyses. All tests took place on the same training court, on the same day of the week, and at similar times. Prior to each test, all players performed a 15-min warm-up routine (consisting of a general cardiac warm-up, specific mobility exercises, and sparring practice).

**TABLE 2:** Prescription of the multifeeding interval training protocols (T_10_, T_30_, and T_50_).

	Number of sets	Intervals per set	Interval duration (s)	Rest between intervals (s)	Interval-rest-ratio	Rest between sets	Active playing time (s)
T_10_	3	20	10	10	1:1	5 min	600
T_30_	2	10	30	30	1:1	5 min	600
T_50_	1	12	50	50	1:1	—	600

### 2.3 Measurements

**FIGURE 2:**
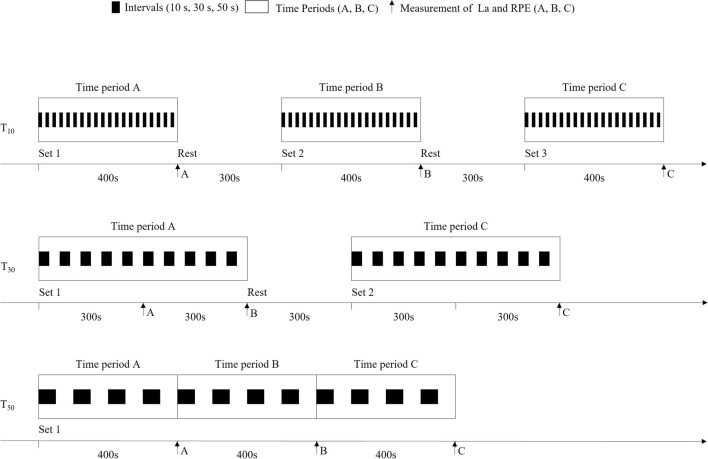
Structure of the protocols (T_10_, T_30_, and T_50_), predefined time points of La and RPE measurements and classification into time periods (A, B, and C).

Ventilation was digitally recorded using Triple-V sensor technology. Inspired and expired air were analysed for oxygen and carbon dioxide concentrations using electrochemical cells and non-dispersive infrared spectrometry, respectively, based on a breath-by-breath approach. Players breathed through a Hans Rudolph mask and carried a mobile spirometry system (MetaMax 3B–R2, firmware version 1.3.40; Cortex Biophysik GmbH, Leipzig, Germany) in a carrying case on their chest. According to previous studies this device provides reliable measurements of metabolic demand with adequate validity for field-based measurements (Vogler et al., 2010). Raw data were transmitted to a remote PC via bidirectional telemetry and stored in the appropriate software (MetaSoft Studio; Cortex Biophysik GmbH, Leipzig, Germany). Breath-by-breath raw data were processed using a moving average of 1 s and exported to the Microsoft Excel software package (Microsoft Office 365; Microsoft Corporation, Redmond, United States). Oxygen consumption (VO_2)_, respiratory exchange ratio (RER), ventilatory exchange ratios, and breathing frequencies were calculated directly from the raw data. Spirometry was calibrated with a 3-L gas flow pump and a standard gas (O_2_, 15.00%; CO_2_, 5.09%) on each test day. HR was recorded using a chest belt (H10 Sensor; Polar Electro GmbH Deutschland, Büttelborn, Germany), which was telemetrically connected to the mobile spirometry device. Gross energy expenditure (EE) was determined via indirect calorimetry. La was determined from capillary blood samples using enzymatic amperometry. Blood samples were taken using 20-µL capillary tubes from the right earlobe at predefined time points (Figure 2). The samples were haemolysed in 1-mL micro test tubes and analysed for La using a Biosen S-Line Sport glucose analyser (EKF-Diagnostik GmbH, Magdeburg, Germany) in the laboratory within 24 h after the testing. Players were asked to scale their RPE, according to Borg (1982), on a scale ranging from 6 to 20 when each blood sample was taken.

Player movements were tracked and measured using an LPS with an integrated accelerometer, gyroscope, and magnetometer (KINEXON ONE, firmware version 1.0; Kinexon GmbH, München, Germany). Sufficient validity (TEE: 1.0%–6.0%) and reliability (CV: 0.7%–5.0%) of the used device for running distances were reported in previous studies (Hoppe et al., 2018). LPS antennae and sensor systems were placed on masts surrounding the playing court. A small, lightweight (14-g) transmitter was fitted between each player’s shoulder blades in a harness supplied by the manufacturer. Using radio-based triangulation (ultra-wideband technology), the sensor system located each player’s position with a sampling rate of 40 Hz in three dimensions (mediolateral, anterior–posterior, and longitudinal). Additionally, nine-axis inertial data were provided with a sampling rate of 200 Hz from the integrated accelerometer. The LPS measurements included distance covered (d), average running velocity (v), and jump frequency (number of jumps per min). The metrics were logged on an open Kinexon interface, transmitted seamlessly to a remote PC, and exported to Microsoft Excel (Microsoft Office 365, Microsoft Corporation, Redmond, United States) for further analysis. For the video recordings, a video camera (LEGRIA HF G3; Canon Deutschland GmbH, Krefeld, Germany) was placed on a balcony behind the court and set in such a way that the whole playing field was visible. Video analyses were used to estimate stroke frequency (SF; number of strokes per s), number of smash shots (sum of ‘smash’ relative to all feeds), and stroke precision. Stroke precision was defined according to the error rate (sum of “missed,” “net,” or ‘out” shots in relation to all shots) and stroke accuracy (relative number of smash shots that were placed in the target zone).

### 2.4 Statistical analyses

Statistical analyses were conducted using Jamovi statistical software (The Jamovi Project, version 1.6). All results are expressed as means ± standard deviations (SDs). Normality was assessed using the Shapiro–Wilk test. In the case of normal distribution, the protocols were compared using repeated analysis of variance (rANOVA) measures. To verify differences over time within each protocol, data were aggregated into equal periods according to the respective protocol structure: T_10_, three periods (A, B, and C) of 200 s; T_30_, two periods (A and C) of 300 s; and T_50,_ three periods (A, B, and C) of 200 s of active playing time, respectively (Figure 2). The rANOVA was used to compare the time periods within each protocol. In case of non-parametric data, the Friedman test was used instead. Paired t-tests (for parametric data) and Wilcoxon tests (for non-parametric data) were used for *post hoc* testing. Range of mean differences (MD) together with smallest lower and highest upper border of the corresponding 95% confidence intervals (95% CI) are reported for significant *post hoc* tastings, respectively. 95% CI is displayed in square brackets as [smallest lower; highest upper]. The significance level was set at 5% (*p* < 0.05) and was adjusted for *post hoc* testing using Tukey correction (p_tukey_ < 0.05). Effect sizes are given as η^2^ and categorised as small (η^2^ = 0.01), medium (η^2^ = 0.06), and large (η^2^ = 0.14) according to Cohen (2013).

## 3 Results

Nineteen players completed all three training protocols. Due to technical failures, some datasets were excluded from the statistical evaluation. Thus, evaluation was based on *n* = 19 for La and video analyses, *n* = 18 for RPE and respiratory measurements, and *n* = 16 for HR and LPS data.

Descriptive and interference statistics for internal training loads are presented in Table 3. The rANOVA revealed no differences in mean and peak VO_2_, HR, or RPE regarding the training protocol. Mean EE did not differ between the protocols, while average peak EE differed between T_10_ and T_30_ [MD (95% CI) = 312 (113; 509) kcal·h^−1^, p_Tukey_ = 0.01]. rANOVA revealed a large effect of interval length on RER and La, respectively. Both increased with increasing interval duration [RER: MD (95% CI) = 0.03–0.06 (0.01; 0.05), p_Tukey_ < 0.01–0.03; La: MD (95% CI) = 1.53–3.23 (0.57; 4.31) mmol·l^−1^, p_Tukey_ < 0.01–0.03]. Gender-related comparisons revealed that male players had higher average and peak VO_2_ (MD = 10.7 mL min^−1^·kg^−1^) and EE (MD = 203 kcal h^−1^) compared to female players, whereas HR, La, and RPE did not differ regarding to gender. Regardless of training protocol and gender, the average HR corresponded to approximately 85% of the individual HR_max_.

**TABLE 3:** Mean and average peak values of internal loads for the three different multifeeding interval training protocols.

		T_10_	T_30_	T_50_	rANOVA	
VO_2_ [ml·min^−1^·kg^−1^]	Mean	45.1 ± 6.0	45.9 ± 7.0	44.0 ± 6.0	*p* = 0.12	η^2^ = 0.02
VO_2_ [ml·min^−1^·kg^−1^]	Peak	69.6 ± 7.0	65.2 ± 8.7	68.6 ± 8.8	*p* = 0.40	η^2^ = 0.04
EE [kcal·h^−1^]	Mean	886 ± 209	919 ± 176	870 ± 206	*p* = 0.04	η^2^ = 0.02
EE [kcal·h^−1^]	Peak	1,660 ± 443^b^	1,363 ± 306^a^	1,472 ± 299	*p* = 0.02	η^2^ = 0.03
RER	Mean	0.90 ± 0.05^bc^	0.93 ± 0.03^ac^	0.96 ± 0.04^ab^	*p* < 0.01	η^2^ = 0.30
RER	Peak	1.19 ± 0.09	1.16 ± 0.07	1.16 ± 0.06	*p* = 0.34	η^2^ = 0.03
HR [bpm]	Mean	164 ± 13	165 ± 11	165 ± 10	*p* = 0.93	η^2^ = 0.00
HR [bpm]	Peak	182 ± 12	183 ± 10	188 ± 12	*p* = 0.08	η^2^ = 0.06
La [mmol·l^−1^]	Peak	3.6 ± 2.0^bc^	5.6 ± 3.0^ac^	7.3 ± 2.3^ab^	*p* < 0.01	η^2^ = 0.25
RPE	Peak	16.1 ± 2.0	17.0 ± 1.7	17.3 ± 1.8	*p* = 0.06	η^2^ = 0.06

Values described as average ± standard deviation; *VO*_*2*_, relative oxygen consumption; *EE*, energy expenditure per hour; *RER*, respiratory exchange ratio; *HR*, heart rate; *La*, blood lactate concentration; *RPE*, rate of perceived exertion; *T*_*10*_, 10 s intervals protocol; *T*_*30*_, 30 s intervals protocol; *T*_*50*_, 50 s intervals protocol.

^a^ significant different to T_10_ (p_tukey_ < 0.05).

^b^ significant different to T_30_ (p_tukey_ < 0.05).

^c^ significant different to T_50_ (p_tukey_ < 0.05).

Overall, players covered 756 ± 168 m (T_10_), 704 ± 130 m (T_30_), and 655 ± 108 m (T_50_) within 600 s of active playing time. While distance covered and average running velocity were comparable for T_10_ (76 ± 17 m min^−1^ and 1.3 ± 0.3 m s^−1^) and T_30_ (70 ± 13 m min^−1^ and 1.2 ± 0.2 m s^−1^), both were significantly lower for T_50_ (65 ± 11 m min^−1^ and 1.1 ± 0.2 m s^−1^) compared to T_10_ [MD (95% CI) = 12 (4; 20) m min^−1^; p_Tukey_ = 0.04]. Players performed fewer jumps in T_10_ (6.7 ± 3.1 min^−1^) compared to T_30_ (8.8 ± 3.8 min^−1^), whereas jump frequency did not differ between T_30_ and T_50_ (8.5 ± 4.2 min^−1^) [MD (95% CI) = 2.9 (1.4; 4.4) jumps·min^−1^, p_Tukey_ < 0.01]. No differences were found for SF (between 0.55 ± 0.06 s^−1^ and 0.58 ± 0.05 s^−1^), error rate (between 16.3% ± 6.0% and 18.7% ± 4.0%), or number of smash shots related to all shots (between 22.4% ± 6.0% and 23.8% ± 7.9%). Descriptive and interference statistics for external loads are presented in Table 4.

**TABLE 4:** Mean and average peak values of external loads and skill performance for the three different multifeeding interval training protocols.

			T_10_		T_30_		T_50_		rANOVA	
Total distance [m]	Sum		756 ± 168^c^		704 ± 130		655 ± 108^a^		*p* = 0.04	η^2^ = 0.05
d [m·min^−1^]	Mean		76 ± 17^c^		70 ± 13		65 ± 11^a^		*p* = 0.04	η^2^ = 0.05
v [m·s^−1^]	Mean		1.3 ± 0.3^c^		1.2 ± 0.2		1.1 ± 0.2^a^		*p* = 0.04	η^2^ = 0.05
Jumps [min^−1^]	Mean		6.7 ± 3.1^b^		8.8 ± 3.8^a^		8.5 ± 4.2		*p* < 0.01	η^2^ = 0.09
SF [s^−1^]	Mean		0.58 ± 0.05		0.57 ± 0.05		0.55 ± 0.06		*p* = 0.08	η^2^ = 0.05
Smash [%]	Mean		22.4 ± 6.0		22.5 ± 6.6		23.8 ± 7.9		*p* = 0.65	η^2^ = 0.01
Error rate [%]	Mean		16.3 ± 6.0		18.7 ± 4.0		18.3 ± 4.0		*p* = 0.12	η^2^ = 0.05
Target zone [%]	Mean		21.9 ± 5.9		23.7 ± 8.2		23.2 ± 8.3		*p* = 0.63	η^2^ = 0.01

Values described as average ± standard deviation; Total distance, accumulated running distance; *d*, running distance per minute; v, running velocity; Jumps, jump frequency per minute; SF, stroke frequency per second; Error rate, number of mistakes (net, out, don`t reach the ball) relative to all shots; Smash, number of smash relative to all shots; Target zone, relative number of smash shots that were placed in hitting zone 4; T_10_, 10 s intervals protocol; T_30_, 30 s intervals protocol; T_50_, 50 s intervals protocol.

^a^ significant different to T_10_ (p_tukey_ < 0.05).

^b^ significant different to T_30_ (p_tukey_ < 0.05).

^c^ significant different to T_50_ (p_tukey_ < 0.05).

Distribution to the hitting zones was independent of the training protocol. Regardless of the protocol, most smash shots were placed in Zone 3 (T_10_, 31.7% ± 7.5%; T_30_, 29.6% ± 8.0%; and T_50_, 32.7% ± 11.1%) and the fewest in Zone 1 (8.6% ± 5.4%, 7.4% ± 6.1%, and 6.0% ± 4.7%). The distribution to error ratios was 18.5% ± 6.8%, 18.8% ± 8.4%, and 18.2% ± 7.8%, the distribution to smash shots that were placed in Zone 2 was 19.3% ± 6.1%, 20.6% ± 8.0% and 19.9% ± 4.6% and the distribution to the target zone was 21.9% ± 5.9%, 23.7% ± 8.2% and 23.2% ± 8.3% for T_10,_ T_30_ and T_50_, respectively. Male and female players did not differ in d, v, jump frequency, SF, number of smash shots, error rate, or stroke accuracy. Descriptive and interference statistics for the performance outcomes are shown in Table 4.

**FIGURE 3:**
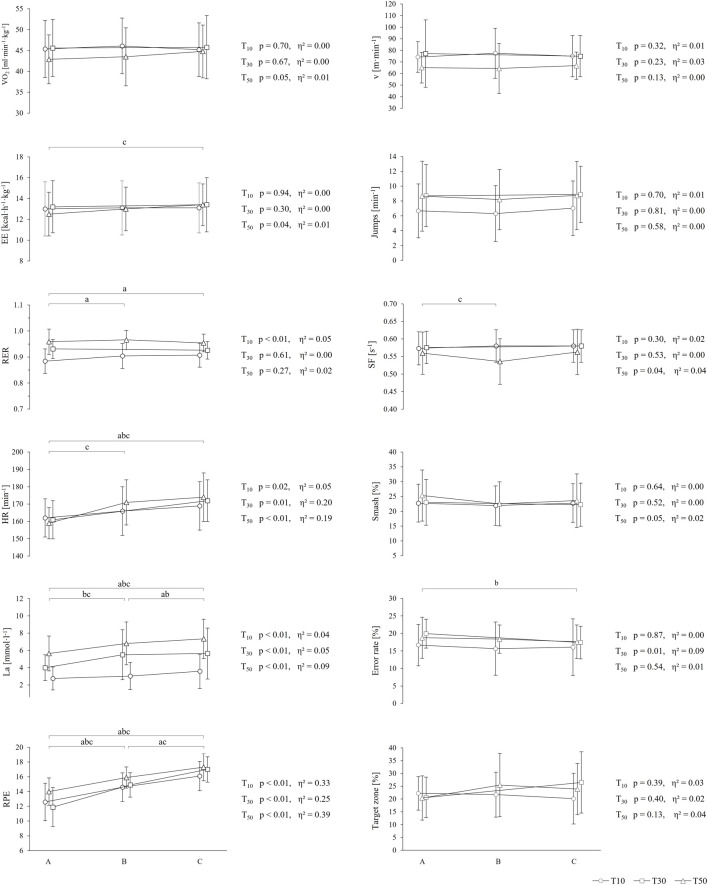
Temporal course of internal loads (*left*), external loads, and performance outcomes (*right*) of the three protocols (T_10_, T_30_, and T_50_). Each point represents the mean ± standard deviation of the predefined time periods (A, B, and C). The presented results of rANOVA show the effect of time periods on the presented parameter, respectively. Significant *post hoc* comparisons are marked as ^a^significant difference within T_10_ (p_tukey_ < 0.05), ^b^significant difference within T_30_ (p_tukey_ < 0.05) and ^c^significant difference within T_50_ (p_tukey_ < 0.05).

Comparing the different time periods (A, B and C) within each training protocol, no significant differences were observed in average VO_2_, for any of the protocols. EE increased in T_50_ from time periods A to C [MD (95% CI) = 0.50 (0.11; 0.89) kcal h^−1^ kg^−1^, p_Tukey_ < 0.01), and RER differed in T_10_ from A to B and A to C [MD (95% CI) = 0.02 (0.01; 0.04), p_Tukey_ < 0.01]. Mean HR, La and RPE increased with increasing drill duration in all protocols. For HR, *post hoc* tests revealed significant differences in T_10_ from period A to C [MD (95% CI) = 6.8 (3.1; 10.6) bpm, p_Tukey_ < 0.01], T_30_ period A to C [MD (95% CI) = 10.2 (5.5; 15.0) bpm, p_Tukey_ < 0.01] and T_50_ period A to B and A to C [MD (95% CI) = 10.3–12.5 (6.6; 16.5) bpm, p_Tukey_ < 0.01]. Post-hoc tests for La showed significance in T_10_ comparing time periods A to C and B to C [MD (95% CI) = 0.55–0.83 (0.18; 1.36) mmol l^−1^, p_Tukey_ = 0.01], T_30_ period A to B, B to C and A to C [MD (95% CI) = 0.13–1.40 (0.31; 2.16) mmol l^−1^, p_Tukey_ < 0.01–0.02], and in T_50_ comparing period A to B, B to C and A to C [MD (95% CI) = 0.53–1.69 (0.01; 2.16) mmol l^−1^, p_Tukey_ < 0.01—< 0.05]. In line with that, *post hoc* tests for RPE showed significant changes between all time periods for all protocols except of T_30_ from B to C [T_10_: MD (95% CI) = 1.5–3.6 (1.0; 4.4), p_Tukey_ < 0.01; T_30_: MD (95% CI) = 2.2–5.0 (1.9; 5.7), p_Tukey_ < 0.01; T_50_: MD (95% CI) = 1.3–3.3 (0.7; 4.4), p_Tukey_ < 0.01]. rANOVA revealed no differences for covered distance, running velocity, jump frequency, number of smash shots, or number of smash shots placed in the target zones. SF decreased significantly in T_50_ from time periods A to B [MD (95% CI) = 0.02 (0.01; 0.03) s^−1^, p_Tukey_ < 0.01] and error rate changed in T_30_ from time periods A to C [MD (95% CI) = 2.8 (1.3; 4.3) %, p_Tukey_ = 0.01]. The temporal courses together with the statistics of rANOVA are illustrated in Figure 3.

## 4 Discussion

The present study compared three different Multifeeding drill protocols that are common in high-performance badminton training practice (10, 30, and 50 s intervals). The results revealed that modifying the interval length led to considerable differences in metabolic response and external training load. Higher La levels highlight the higher reliance on anaerobic energy metabolism during longer intervals compared to shorter intervals, while average oxygen uptake did not differ between the different protocols. Additionally, with longer interval length total distance covered and average running velocity decreased. Although no reduction in stroke precision was observed, a reduction in stroke velocity seemed likely. The findings demonstrate the importance of evidence-based training control that is orientated on training goals.

Multifeeding drills are typically used as interval based on court training to enhance the badminton-specific endurance capacity. Considering the specific requirements of the sport, this mainly means improved aerobic capacity, efficient La elimination, faster reoxygenation of myoglobin, and greater resynthesis of muscular energetic phosphates (ATP and CrP) (Liu et al., 2021). In competitive match play, rallies can last between 1 and 40 s, but analysis of the frequencies showed that most rallies (47%) last between 3 and 6 s, and only 13% of all rallies exceed a 9-s duration (Faude et al., 2007). Even in tournaments at the highest international level (e.g., the Olympic Games), peak durations do not exceed 45 s (Torres-Luque et al., 2019). Accordingly, the external loads of, and consequently the physiological responses to, the 10 s training intervals most closely represented the average loads of competitive match play, whereas the 30 s intervals reflected the peak anaerobic loads, and the 50 s protocol led to an anaerobic load that might be achieved only occasionally, if at all [Average loads during match play: VO_2_, 46.0–55.7 mL min^−1^ kg^−1^; HR, 166–179 bpm; La, 1.9–4.7 mmol l^−1^ (Edel et al., 2019)]. The present study revealed an average VO_2_ of approximately 45 mL min^−1^ kg^−1^ and a mean HR about 165 bpm, which corresponded to 85% of HR_max_, regardless of the protocol (Table 3). This points to the high load on the aerobic and cardiovascular systems in Multifeeding drill practice. However, no differences in the mean and peak VO_2_ or HR were observed between the protocols and current literature considers a mean HR of 85% HR_max_ sufficient to enhance specifically required abilities in badminton players (Phomsoupha et al., 2019). Consequently, all the considered protocols seemed to provide an effective training stimulus for aerobic metabolism. In contrast, average RER (T_10_, 0.90 ± 0.05; T_30_, 0.93 ± 0.03; T_50_, 0.96 ± 0.04) and La (T_10_, 3.6 ± 2.0; T_30_, 5.6 ± 3.0; T_50_, 7.3 ± 2.3 mmol l^−1^) increased with increasing interval duration (Table 3). This indicates a higher utilisation of anaerobic lactic pathways in relation to the length of the intervals, which is in line with general findings for intermittent exercises (Latzel et al., 2018).

Interestingly, the differences in anaerobic metabolic loads were not related to differences in the perceived level of exertion, as players reported comparable RPE for all three protocols (Table 3). In line with that, Phomsoupha et al. (2019) supposed that RPE might be rather related to attentional and neuromuscular than metabolic fatigue and concluded that the overall workload in badminton is determined more by the number and exertion of numerous high-intensity actions (such as strokes, jumps, accelerations, and decelerations) than by the duration of rallies (Phomsoupha et al., 2019). Therefore, one possible explanation why longer interval durations were not perceived as more strenuous might be that players used compensating pacing strategies to regulate exercise intensity.

During competitions, players typically influence the workload by reducing the SF through an adjustment of shuttlecock trajectory and stroke velocity and prolonging or shortening of recovery phases (Phomsoupha et al., 2019). However, the study design assumed a consistent serving frequency (with no breaks and in random order) and a predetermined rest duration between intervals. Therefore, the tactical behaviour of the players could not affect the training intensity in this setup. However, SF and number of smash shots (which were expected to be the most effortful strokes (Phomsoupha et al., 2019) did not differ between the protocols (Table 4), but was considerably lower than typically observed under match-play conditions (0.92–1.09 s^−1^) (Edel et al., 2019). Considering that the work-to-rest ratio in this investigation (1:1) was higher than during match play (1:2) (Faude et al., 2007), the likely explanation for the lower SF might be that coaches adjusted the serve frequency in a way that allowed the players to maintain training intensity despite the proportionally shorter rest times.

From a physiological point of view, a reduction in exercise intensity in the case of longer intervals could be expected, since muscular acidosis inhibits the enzymatic (phosphofructokinase) processes that are responsible for the glycolytic flux rate (Leite et al., 2011). Accordingly, distance covered and average running velocity in the 50 s intervals were significantly lower compared to the 10 s intervals (Table 4). These findings, together with a comparable average SF, suggest that either the coaches adjusted the serves to make it easier (with shorter steps) to reach the shuttlecocks, or that the players reduced their running paths, bearing the cost of being in a less favourable position for the stroke, as a shorter running path predicts a greater distance to the shuttlecock trajectory, which necessarily leads to a lower stroke height and impairs the efficiency of the proximo-todistal force transmission from a biomechanical point of view (Rusdiana et al., 2020). This probably results in a poorer performance, in terms of a later contact point and lower stroke velocity, which are main determinants for putting pressure on the opponent and for placing oneself in a better position (Rusdiana et al., 2020). However, for practical reasons, stroke height and velocity could not be measured in the present investigation.

Surprisingly, movement tracking revealed that the number of jumps was higher for the 30 s than the 10 s intervals, indicating an increase in intensity in the case of the longer intervals (Table 4). However, this trend disappeared when the intervals were extended to 50 s. Therefore, a possible explanation for these findings might be a temporal constraint caused by the study design. In the present investigation, the players had to start each interval from the back or side of the court. Therefore, the first serve was not played as a jump smash for practical reasons. In summary, a total of 60 intervals in the 10 s protocol compared to a total of 20 intervals in the 30 s protocol might have led to cumulative measurement error and explained the differences. Moreover, although the number of intervals (12) was even lower in the 50 s protocol, no differences were observed compared to the 10 s and 30 s protocols. Therefore, a reduction in jump frequency probably occurred for the 50 s intervals but was missed due to the cumulative measurement error. In addition, number of jumps is also affected by the way the coaches serve the shuttlecocks. Thus, further studies should consider a more standardized serving as well as an equal number of intervals to clarify this issue.

In the present study, the quality of performance outcomes was determined by stroke precision (error rate and stroke accuracy). Previous research showed that the performance outcomes in team and racquet sports depend greatly on the physiological strain caused by short-term, high-intensity activities during intermittent exercise. In this context, for badminton, increasing fatigue was found to relate to reduced stroke velocity and accuracy due to decreased joint velocity in the proximo-to-distal sequencing (Rusdiana et al., 2020), a poorer handgrip (Phomsoupha et al., 2019), and a reduced level of neuronal activation (Le Mansec et al., 2020). Moreover, an increased level of acidosis was related to a decrease in coordinative abilities and hand–eye coordination (Majumdar et al., 1997; Ceylan et al., 2016). Therefore, poorer performance outcomes due to greater anaerobic loads could be expected in the case of longer intervals. However, this study did not reveal any differences in error frequency or stroke accuracy (number of smash shots placed in target zone) relative to interval length (Table 4). According to Missenard et al. (2009), who showed that the motor system adapts motor planning and execution to fatigue by reducing movement velocity to preserve movement accuracy, one very likely explanation for the current findings may be that players accepted reduced stroke velocity to maintain stroke precision.

Considering the temporal courses within each training session, during all protocols, the mean VO_2_ remained constant, while the HR and RPE continued to increase. The high HR may have resulted from cardiac drift, and the simultaneous increase in RPE possibly indicates increased mental fatigue. In addition, La levels increased during the drill, suggesting a constantly high La accumulation during the rallies and that the 1:1 interval-to-rest ratio was inadequate for La removal (Figure 3). The increasing acidosis suggests that players were forced to reduce the training intensity by reducing the external load or accepting worse performance outcomes during the drill. However, enhanced internal loads were not related to reduced average running velocity, number of jumps, or relative number of smash shots, but in the case of the 50 s protocol, reduced SF was observed between the first and second thirds of the training drill, which was compensated for in the last third. Despite the expectation of constant SF, the influence of the players’ fatigue on the serving frequency of the feeder cannot be excluded. Lastly, no differences in stroke accuracy could be found, but the error frequency decreased between the first and second halves of the 30 s protocol (Figure 3). This might be attributable to a coordinative improvement in terms of a warmup effect being superior to the metabolic fatigue.

To the best of our knowledge, this was the first investigation to compare different badminton on-court drill training protocols. An early study by Majumdar et al. (1997) investigated physiological responses to Multifeeding drills (intervals of 40–50 s, with rest times of 60–120 s). They reported comparable VO_2_ (45 mL min^−1^ kg^−1^), HR (between 82% and 100% of HR_max_), and La (between 8.0 and 10.5 mmol l^−1^) and concluded that the anaerobic metabolic load of training drills may be too high to maintain performance outcomes. However, they did not consider variations in the training regimes, so this study did not allow conclusions to be drawn regarding the impact of interval length. Another training study compared different rest times in a tennis-specific passing-shot drill (Ferrauti et al., 2001b). In this study, shorter rest times led to higher muscular acidosis, which resulted in decreased v, accompanied by shorter running paths. Moreover, the researchers demonstrated that the shorter running path paralleled a considerable reduction in stroke velocity and assumed that an increased level of fatigue led to changes in stroke intention (avoiding errors vs. playing successful attacking shots) (Ferrauti et al., 2001b). Accordingly, although stroke velocities were not measured in the present investigation, due to the higher acidosis, the resulting poorer positioning, and comparable results for SF and stroke precision, reduced stroke velocity in the case of longer intervals seems very likely.

### 4.1 Limitations

To provide results with high practical relevance, the present study was designed to examine high-performance badminton training practice. However, this involved some limitations. Firstly, the findings suggest that coaches and players used compensating pacing strategies to ensure the maintenance of high internal and external loads during the drill. As coaches and players knew the duration and total number of the intervals, enhanced motivation during the last few intervals of the drills and during the last seconds of each interval could not be excluded. This could have led to reduced pacing and greater player effort towards the end of each drill or interval, which could have masked changes in external loads or performance outcomes across the training drill. Moreover, to maintain the total duration of the drills, the number of intervals had to be adjusted for each protocol. Therefore, a practical limitation of the study is that players had to start each interval either from the side or the back of the court, which might have influenced the distribution of the stroke techniques at the beginning of each interval. The different numbers of intervals could have caused cumulative measurement error in the notational and kinematic analyses. Lastly, this study assumed a reduction in stroke velocity due to poorer strike positions and the maintenance of stroke precision. However, stroke velocity could not be measured as the study design allowed players to strike from any position on the court, and this setup would cause a high angular error in measurements taken via a speed gun positioned behind the court. Further studies could reveal the impact of interval duration and anaerobic load on performance outcomes under more standardised conditions. Moreover, the present study reveals only an acute observation. Further research could also analyse the impact on specific in-game performance outcomes after a training intervention with these different protocols over an extended period (e.g., for several weeks).

### 4.2 Practical recommendations

In general, training prescriptions should be oriented towards the competition requirements of a sport. From a scientific point of view, this means that medium, average peak, and maximum loads should be justifiable in training practice. All the investigated variations (10, 30, and 50 s) are used regularly in high-performance badminton training. In this context, the 10 and 30 s protocols were assumed to represent the average and peak match play conditions, respectively, whereas the 50 s protocol could be rated as a supramaximal load. Usually, practitioners justify longer intervals with improved La tolerance and mental competitiveness. However, the 50 s protocol led to La levels that are rarely achieved during competitions, as players avoid such high muscular acidosis by tactically prolonging of rest periods. Moreover, players reported comparable RPE for all three protocols, suggesting that the perceived mental effect might also be negligible. Regarding the decline in exercise intensity and the potential decrease in stroke velocity that accompanies higher acidosis, it is questionable whether extending the interval length beyond 30 s in this training setting could further improve the specific training adaptations. To incorporate supramaximal loads into training, the 50 s intervals should be included as single peaks or performed for shorter total durations instead, to allow players to maintain a maximum trainings intensity.

## 5 Conclusion

Comparing different interval lengths in badminton Multifeeding training revealed that in the case of longer interval durations, anaerobic metabolic stimulus increases while distance covered and average running velocity decrease. Even though there was no impact on stroke precision, extending the intervals beyond 30 s might decrease the external training load and impair performance outcomes. Therefore, the interval duration should be defined carefully according to the training goals.

## Data Availability

The raw data supporting the conclusion of this article will be made available by the authors, without undue reservation.
